# Editorial: Natural language processing for recommender systems

**DOI:** 10.3389/fdata.2025.1573072

**Published:** 2025-03-25

**Authors:** Alfred Krzywicki, Michael Bain, Wayne Wobcke

**Affiliations:** ^1^School of Computer Science and Mathematics, University of Adelaide, Adelaide, SA, Australia; ^2^School of Computer Science and Engineering, University of New South Wales, Sydney, NSW, Australia

**Keywords:** natural language processing (NLP), recommender systems, large language models, hybrid recommender systems, contextual recommender systems

## Introduction

Recommender systems have become indispensable in the information age, guiding users through vast datasets and enabling personalized, contextually relevant interactions. By leveraging user and item similarities through collaborative filtering and content-based strategies, these systems aim to match user preferences with novel and useful suggestions. Textual data, rich in meaning, has been key to this progress, with recent advances in machine learning and NLP making it even more useful. The advent of Large Language Models (LLMs) has enabled deeper understanding of context and semantics, transforming how text informs recommendations.

The four articles in this Research Topic show how NLP is used in recommender systems to solve different challenges and improve modern methods. They highlight how NLP can enhance systems in areas like data analysis, user satisfaction, skill evaluation and language translation.

Bhuvaneswari and Varalakshmi propose a novel hybrid framework that integrates Neural Machine Translation (NMT) and Statistical Machine Translation (SMT) for improving translation performance in low-resource language pairs, specifically English-Tamil.

This research aligns with prior studies like Lample et al. (2018) and Qi et al. (2018) in leveraging monolingual data for low-resource translation. However, unlike these studies that primarily rely on back-translation, the hybrid NMT-SMT approach optimizes translation quality by systematically selecting high-quality outputs. The use of beam search decoding, as supported by Freitag and Al-Onaizan (2017), further enhances the model's efficacy.

Compared to Wang et al. (2017), who explored phrase-based SMT and reranking for higher-resource languages, this paper extends the application to low-resource settings, addressing unique challenges like idiomatic expressions and rare word handling. The integration of SMT ensures better initialization and incremental improvement, demonstrating a novel contribution in advancing machine translation for under-resourced languages.

Dietz et al. present a comprehensive study on data-driven models for travel destination characterization in recommender systems. The research addresses the challenge of selecting data sources and features that best align with the concept of a “touristic experience,” which lacks a clearly defined ground truth.

This study complements existing research such as Quercia et al. (2015), which also explores data-driven models for urban analytics. Unlike these studies, Dietz et al. focus on a systematic evaluation of methods using rank agreement metrics and expert validation, offering a unique perspective on optimizing recommender systems. Compared to similar works in content-based recommendation, such as Lops et al. (2011), this paper emphasizes the integration of textual data and expert-grounded evaluation, advancing practical applications in tourism.

Jemal et al. introduce a multi-modal recommender system designed to predict project manager performance within a competency-based framework. The research focuses on automating competency score prediction to address the inefficiencies and biases inherent in manual assessment.

This work aligns with performance modelling approaches, such as Thai-Nghe et al. (2010) in education-focused systems, but extends them to project management with a multi-modal and NLP-enhanced framework. Unlike existing models such as Shahhosseini and Sebt (2011), which use fuzzy logic to assign competencies in construction projects, Jemal et al. incorporate robust recommendation techniques and NLP embeddings to enhance prediction accuracy.

The study's focus on multi-modal data integration sets it apart from traditional frameworks (e.g., Dainty et al., 2005), while its use of advanced NLP tools contrasts with simpler regression-based methods. By addressing cold-start challenges for new users and competencies, this research makes a significant contribution to both recommender systems and competency-based evaluation.

Zhang et al. investigate the effects of feature-based explanations and output modalities (text vs. voice) on user satisfaction with service recommender systems.

This study aligns with findings by Tintarev and Masthoff (2012) and Bilgic and Mooney (2005) on the importance of explanations for transparency and user trust. However, it diverges by highlighting the nuanced role of modality, an area less explored in previous work like that of Herlocker et al. (2000) or Chen and Pu (2005). Unlike Kouki et al. (2019), who focused on persuasiveness, Zhang et al. provide empirical evidence on satisfaction variability by modality and design factors, extending the applicability of explanations in service domains.

## Discussion

These studies share several common themes that highlight key priorities and methods in using NLP for recommender systems. First, all emphasize the importance of context. Whether understanding data, explaining recommendations or evaluating competencies, context helps make recommendations more relevant and useful.

Second, the studies use advanced NLP techniques to analyse and transform text data. For example, Dietz et al. use ranking methods, while Bhuvaneswari and Varalakshmi rely on hybrid training models. These approaches show how NLP not only supports but also drives solutions for specific challenges, delivering clear performance improvements.

Third, there is a focus on innovation through combining different methods and data types. Jemal et al. use a multi-modal framework, while Zhang et al. explore how different explanation styles affect user satisfaction. These examples show the growing need for more complex systems that can handle diverse requirements, which aligns with the trend of using hybrid models and multi-modal data processing to improve recommender systems.

## References

[B1] Bilgic M. Mooney R. J. (2005). “Explaining recommendations: satisfaction vs. promotion,” in Beyond Personalization Workshop, IUI'05, 13–18.

[B2] Chen L. Pu P. (2005). “Trust building in recommender agents,” in Proceedings of the Workshop on Web Personalization, Recommender Systems and Intelligent User Interfaces at the 2nd International Conference on E-Business and Telecommunication Networks, 135–145. 10.5220/0001422901350145

[B3] Dainty A. R. Cheng M. I. Moore D. R. (2005). Competency-based model for predicting construction project managers' performance. J. Manag. Eng. 21, 2–9. 10.1061/(ASCE)0742-597X(2005)21:1(2)

[B4] Freitag M. Al-Onaizan Y. (2017). Beam Search strategies for neural machine translation. arXiv preprint: arXiv:1702.01806.

[B5] Herlocker J. L. Konstan J. A. Riedl J. (2000). “Explaining collaborative filtering recommendations,” in Proceedings of the 2000 ACM Conference on Computer Supported Cooperative Work, 241–250. 10.1145/358916.358995

[B6] Kouki P. Schaffer J. Pujara J. O'Donovan J. Getoor L. (2019). “Personalized explanations for hybrid recommender systems,” in Proceedings of the 24th International Conference on Intelligent User Interfaces, 379–390. 10.1145/3301275.3302306

[B7] Lample G. Ott M. Conneau A. Denoyer L. Ranzato M. (2018). Phrase-based and neural unsupervised machine translation. arXiv preprint: arXiv:1804.07755.

[B8] Lops P. de Gemmis M. Semeraro G. (2011). “Content-based recommender systems: state of the art and trends,” in Recommender Systems Handbook, 73–105. 10.1007/978-0-387-85820-3_3

[B9] Qi Y. Sachan D. S. Felix M. Padmanabhan S. J. Neubig G. (2018). When and why are pre-trained word embeddings useful for neural machine translation? *arXiv preprint: arXiv:1804.06323*.

[B10] Quercia D. Schifanella R. Aiello L. M. McLean K. (2015). “Smelly maps: the digital life of urban smellscapes,” in Proceedings of the 9th International AAAI Conference on Web and Social Media, 327–336. 10.1609/icwsm.v9i1.14621

[B11] Shahhosseini V. Sebt M. H. (2011). Competency-based selection and assignment of human resources to construction projects. Scient. Iranica 18, 163–180. 10.1016/j.scient.2011.03.026

[B12] Thai-Nghe N. Drumond L. Krohn-Grimberghe A. Schmidt-Thieme L. (2010). Recommender system for predicting student performance. Proc. Comput. Sci. 1, 2811–2819. 10.1016/j.procs.2010.08.006

[B13] Tintarev N. Masthoff J. (2012). Evaluating the effectiveness of explanations for recommender systems: methodological issues and empirical studies on the impact of personalization. User Model. User-Adapt. Inter. 22, 399–439. 10.1007/s11257-011-9117-5

[B14] Wang X. Lu Z. Tu Z. Li H. Xiong D. Zhang M. (2017). “Neural machine translation advised by statistical machine translation,” in Proceedings of the Thirty-First AAAI Conference on Artificial Intelligence. 10.1609/aaai.v31i1.10975

